# Physiological linkage of thyroid and pituitary sensitivities

**DOI:** 10.1007/s12020-022-03184-8

**Published:** 2022-09-17

**Authors:** Stephen Paul Fitzgerald, Nigel G. Bean, Henrik Falhammar, Rudolf Hoermann

**Affiliations:** 1 The Departments of General Medicine and Endocrinology, The Royal Adelaide Hospital, Adelaide, SA Australia; 2 The University of Adelaide, School of Medicine, Adelaide, SA Australia; 3 School of Mathematical Sciences and ARC Centre of Excellence for Mathematical and Statistical Frontiers, University of Adelaide, Adelaide, SA 5000 Australia; 4 Department of Endocrinology, Karolinska University Hospital, 171 76 Stockholm, Sweden; 5 Department of Molecular Medicine and Surgery, Karolinska Institutet, 171 76 Stockholm, Sweden; 6 Klinikum Lüdenscheid, Paulmannshöherstr. 14, 58515 Lüdenscheid, Germany; 7 113 Andersons Road, Yandina, QLD 4561 Australia

**Keywords:** Thyroid physiology, Thyroid function, Thyrotropin (TSH), Thyroid hormones

## Abstract

**Objectives:**

The sensitivities of the pituitary to thyroxine feedback, and the thyroid to thyrotropin stimulation determine the free thyroxine /thyrotropin feedback loop and can be described mathematically by two curves. It is not well understood how the two curves combine in a healthy population with normal thyroid function to express the individual balance points that are observed. This study was directed at this issue testing the possibilities of random combination and directed linkage between the two curves.

**Methods:**

We reverse-engineered two sets of population data, on the assumption of independent combinations of thyroid and pituitary sensitivities, to obtain estimates of the curve describing thyroid sensitivity. Sensitivity studies were performed.

**Results:**

No analysis resulted in a physiologically feasible estimate of the curve describing thyroid sensitivity. There was evidence of linkage of the two curves in terms of their combination throughout the normal range. Thyroid response curves reflecting a low free thyroxine response to thyrotropin tended to be combined in individuals with thyrotropin curves reflecting a high thyrotropin response to free thyroxine, and vice versa.

**Conclusions:**

Thyroid and pituitary sensitivities are linked, being combined in individuals in a non-random directed pattern. Direct mutual interaction may contribute to this linkage. This linkage precludes the derivation of the curves describing these sensitivities from population data of the free thyroxine and thyrotropin relationship and complicates their derivation by physiological experimentation. This linkage and probable interaction may also bestow evolutionary advantage by minimising inter-individual variation in free thyroxine levels and by augmenting homeostasis.

## Introduction

Thyroid function, and consequently thyroid hormone levels, are regulated predominantly by feedback control from the hypothalamus-pituitary [1]. Thyrotropin (TSH) released from the pituitary thyrotropes, under the influence of thyrotropin-releasing hormone (TRH) from the hypothalamus, stimulates the production of thyroid hormones, principally thyroxine (T4) and triiodothyronine (T3), by the thyroid gland [2, 3]. The free levels of these hormones in the circulation, free thyroxine (FT4) and free triiodothyronine (FT3), affect via intracellular T3 the function of many if not most organs and tissues in the body, directly and via effects on the brain [1, 4, 5]. With two separate feed-back loops, FT4 and FT3 also act on the hypothalamus and the pituitary to decrease the release and circulating levels of TSH [1]. Feedback is mainly a FT4 mediated mechanism [1] at least partially on the basis that intracellular monodeiodination of circulating FT4 is a substantial contributor of pituitary nuclear T3 [1, 6]. Population data correspondingly show a stronger negative correlation between FT4 and TSH than between FT3 and TSH [7].

Beyond this basic outline of the predominant mechanism of thyroid regulation there are other mechanisms at play such that thyroid regulation is multi-faceted. These mechanisms include an ultra-short feedback loop whereby TSH suppresses its own release [8–10] and possibly a feed-forward mechanism by which TSH increases de-iodinase activity [8].

As T4 is the main thyroid hormone secreted by the thyroid in response to TSH and is the circulating hormone best correlated with TSH suppression and with the peripheral tissue and organ thyroid status [11] we devoted this paper to FT4 and TSH regulatory physiology. We were cognisant of the myriad of receptor and metabolic factors which modulate the above physiology centrally and peripherally [1, 12] but considered that these factors did not affect our enquiry.

Physiologically, the control mechanisms of the hypothalamic-pituitary thyroid (HPT) axis are very strong. Normal levels of FT4 bring the uninhibited TSH observed in a hypothyroid state down by a factor of 500 to 1,000 (500 to 1,000 mIU/L to 1 mIU/L), and TSH raises hypothyroid FT4 levels (approximately 1 pmol/L), which exist in the absence of any TSH stimulation to those in the euthyroid state (approximately 15 pmol/L) [13]. Each of these processes can be described mathematically as curves characteristic of the pituitary and the thyroid [1]. The pituitary TSH response to FT4, the sensitivity of the pituitary, is described by the ‘TSH curve’ and the thyroid FT4 response to TSH, the sensitivity of the thyroid, is described by the ‘FT4 curve’. Normal thyroid function is dominated by the interplay of these two curves and the normal population range of thyroid function reflects the normal range of these curves [1].

**Fig. 1:**
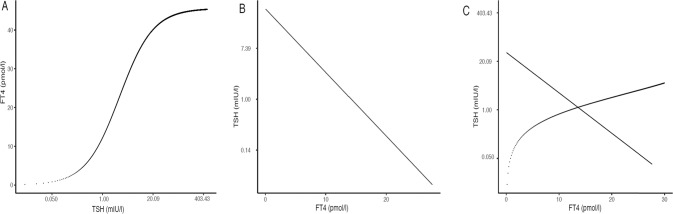
The FT4 curve, the TSH curve, and the derivation of an individual equilibrium FT4/TSH point at the intersection of the 2 above curves. The FT4 curve (**A**), the TSH curve (**B**), and the derivation of an equilibrium FT4/TSH point at the intersection of these 2 curves (**C**)

**Fig. 2:**
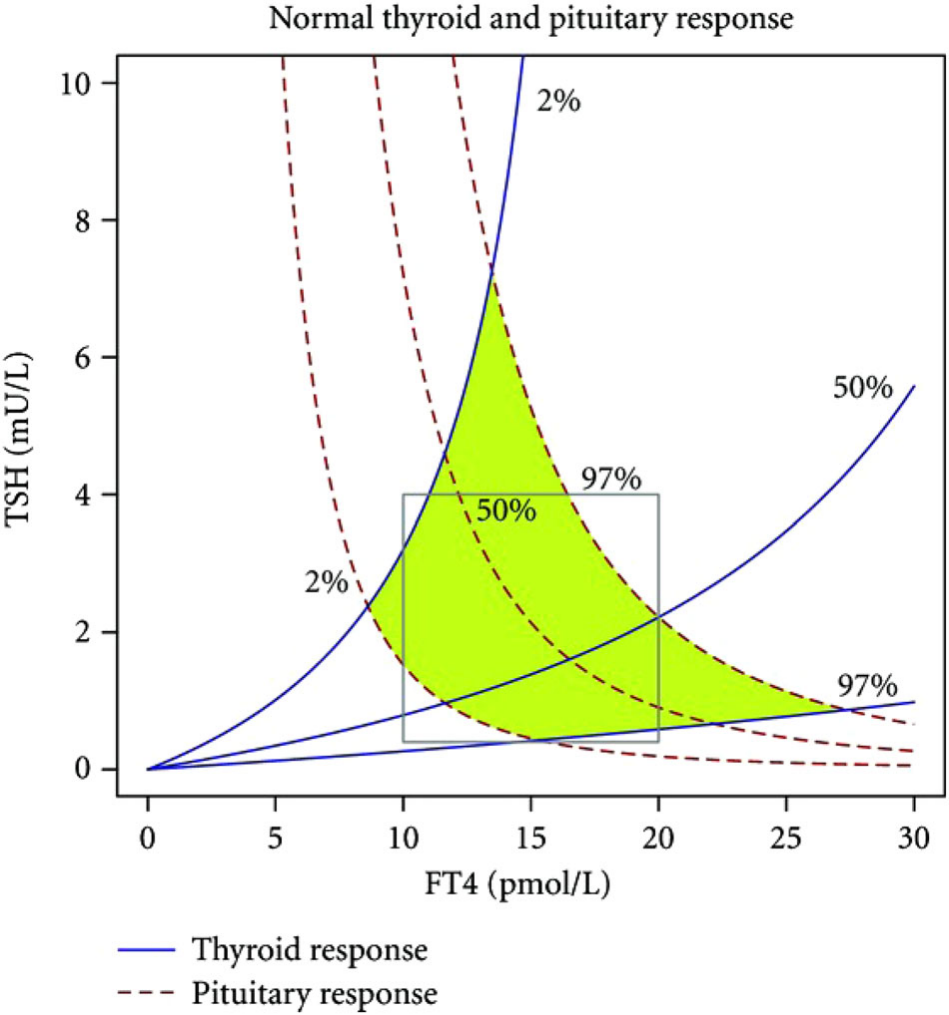
[1] The range of normal FT4 and TSH curves. Individuals’ balance points sit at intersections of the various curves. The conventional normal range defined by normal FT4 and TSH values is delineated by a corresponding rectangle, The green kite-shaped area indicates a range defined by normal FT4 and TSH curves

Figure 1 shows schematically these curves for an individual as well as the derivation of the individual’s FT4/TSH balance point (often termed ‘set point’), i.e., the intersection of these 2 curves. Figure 2 shows inter-individual variation in the FT4 and TSH curves, reflecting inter-individual variation in thyroid and pituitary sensitivities, the consequent variation in the individual combinations of the two curves and thereby the inter-individual variation in the FT4/TSH balance point. Though normal thyroid function is usually defined by separate reference ranges of thyroid hormone and TSH values, the ‘kite shaped’ (quadrilateral shaped if y axis is in log) area bounded by centile TSH and FT4 curves provides an alternative inter-related way of defining normal thyroid physiology (Fig. 2) [1].

Though there are many potential sources of intra-individual variation in thyroid function [14], FT4 and TSH levels tend to be relatively stable for each healthy individual in the population such that inter-individual variation, as manifest as the population range, is greater than intra-individual variation [15]. This stability in turns implies intra-individual stability of thyroid and pituitary sensitivity.

The definition of the TSH and FT4 curves has not been easy. Experimental data from individuals on thyroxine replacement/over-replacement has indicated that the relationship between TSH levels and FT4 levels is log-linear [16–19]. Though these studies have all provided log-linear curves they have differed from each other and furthermore may not represent physiology exactly given that the individuals were not normal in that they were hypothyroid, and/or given that thyroxine replacement/supplementation therapy is not strictly physiological.

As difficult as defining the TSH curve has been, the empiric definition of the FT4 curve has been even more challenging. It is not currently feasible to study individuals with thyrotrope insufficiency by replacing TSH physiologically in order to observe the FT4 response.

There has been less study as to how the physiological curves may be combined in individuals. It has previously been thought that there is a ‘set point’ of thyroid hormone levels [1, 12]. A ‘set point’ implies that encoded somewhere in the body there is a reference or ideal value of a given parameter against which ambient levels are compared. In the event of the ambient level differing from this encoded value corrective physiological mechanisms are activated to restore the set point level [20]. In these circumstances FT4 curves would be combined with TSH curves in individuals as required, i.e. pituitary sensitivity to FT4 would be adjusted to achieve the desired set point FT4 level [21].

However, we have shown that the population data of FT4/TSH provides evidence against there being such a set point for thyroid function and that rather thyroid hormone values represent ‘balance points’ [21]. A ‘balance point’ implies that the level of a given parameter is merely the consequence of the various physiological processes relevant to that parameter [22].

The consideration of normal thyroid function on the basis of normal FT4 and TSH curves as above [1] implies a random combination of FT4 and TSH curves e.g. all individuals on the 97th TSH percentile are normal provided this curve is combined with an FT4 curve between the 2nd and 97th centiles.

**Fig. 3:**
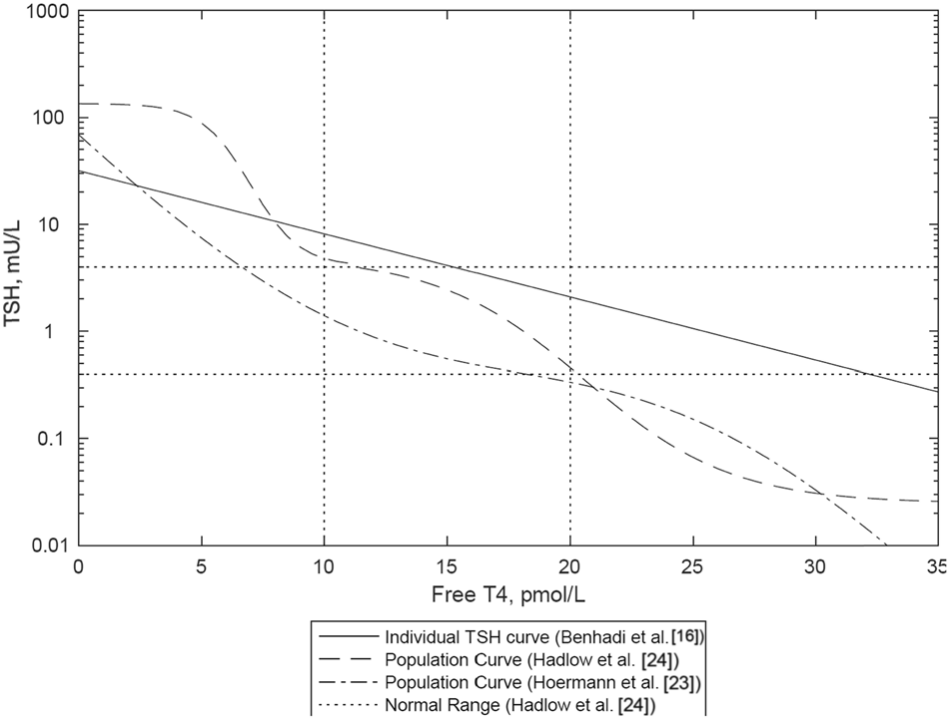
[26] Two population curves together with the TSH curve estimated physiologically by Benhadi

In addition to the above-mentioned physiological studies, there have been attempts to infer the TSH curve from the population data of paired FT4/TSH values by the analysis of the slope of the correlation between FT4 and TSH (‘the population curve’) [23, 24]. These studies provided a somewhat similar estimate of the shape of the TSH curve, but the derived curves were not strictly log-linear (Fig. 3). Hoermann et al. [23] indicated that the slope may increase with deviation from an optimum set point (sic) and that on account of interindividual variation the population curve does not necessarily reflect the TSH response curve of all individuals [25].

We have further demonstrated [26] that the population distribution of FT4/TSH rather than being a general reflection of the TSH curve is a function of the relative interindividual variation in the distributions of TSH and FT4 curves and the manner of combination of the two curves in individuals. Only in the context of random, independent combination can the slope of the population curve match the slope of the TSH curve.

In particular we reported that in the presence of a linkage between the curves such that thyroids of low sensitivity were preferentially combined with pituitaries of low sensitivity, the slope of the population curve may be flatter than the true physiological TSH curve [26]. However, given the similarity of slope in the normal range of one of the population curves [23] to the one of the experimentally derived TSH curves [16] (Fig. 3) we concluded that these two curves may be the most accurate of those proposed and that FT4 and TSH curves combined randomly [26]. We further concluded that the negative slope of the population data was therefore likely to be due to greater interindividual variation in FT4 curves than in TSH curves [26].

On the other hand, Hoermann et al. have reported evidence of non-random combination of thyroid and pituitary sensitivities [27]. The emphases in their study were on the shifts seen in pituitary function with thyroid gland disease and the possible modulating role of FT3, rather than on the significance of this type of combination within the normal range. In their study the non -random combination did nevertheless extend from the low normal range of individuals with mild thyroid dysfunction through the normal range.

The conclusions of this study by Hoermann et al. are however limited by the methodology used. To assess pituitary function, the authors used the TSH Index (TSHI) formula which, though able to predict other pituitary dysfunction [28] relies on the slope of the data to estimate the slope of the TSH curves [28], thereby not accounting for any error introduced by their demonstrated non-random combinations of FT4 and TSH curves [27].

Hoerman et al. [27] confirmed their findings also using the Thyrotroph Thyroid Hormone Resistance Index (TTSI). This index however was originally used in the assessment of thyroid resistance syndromes [29, 30] rather than for normal individuals and furthermore as it is based on the product of TSH and FT4 levels, it relies on there being identical but reciprocal changes in TSH levels for any change to FT4 levels for any given degree of thyroid resistance. It thus does not accurately describe the relationship of pituitary sensitivity as determined physiologically.

In this paper we describe our further work in this area. We reasoned that if FT4 and TSH sensitivities are combined randomly to generate individual FT4/TSH levels it should be possible to derive from the population FT4/TSH data not only an estimate of the TSH curve but also an estimate of the FT4 curve. We are not aware of any previous attempts to derive the FT4 curve from population data of paired FT4/TSH values.

## Methods

We studied two population distributions of FT4/TSH, one from a general German population and another from a population of only ostensibly normal subjects not on thyroid replacement. The general population sample included 1424 individuals from a previous study [23], excluding known thyroid, hypothalamic or pituitary pathology, overt non-thyroidal illness, pregnancy and subjects under the age of 18. Parameters of this sample included median age 62 (range 18–98), mean FT4 13.2pmol/L (2.93), and median TSH 1.01mIU/L (interquartile range 0.44:1.81). The ostensibly normal subject group included 450 clinically euthyroid individuals from another, previous study [31]. This latter population was prospectively sampled, well characterised and carefully selected on the basis of an unremarkable detailed patient history including absence of, thyroid disease, comorbidity with nonthyroidal illness including pituitary disease and pregnancy, medication including thyroid hormone replacement and any thyroid-associated symptoms, and a clinical examination indicating euthyroidism. Normal laboratory tests for inclusion included FT3, FT4, and TSH, and, to rule out autoimmune thyroiditis, thyroid peroxidase antibodies (TPO-Abs) and/or TSH receptor antibodies (TSH-R Abs). Thyroid ultrasound was routinely performed on all patients, followed by thyroid scintigraphy in some cases with larger nodules >1 cm to rule out toxic adenoma. Parameters of this sample included median age 44.4 (5–95), female 70.8%, FT4 14.3pmol/L (1.77), TSH 1.43 mIU/L (0.94:2.06), and thyroid volume 14.8 ml (9.55).

**Fig. 4:**
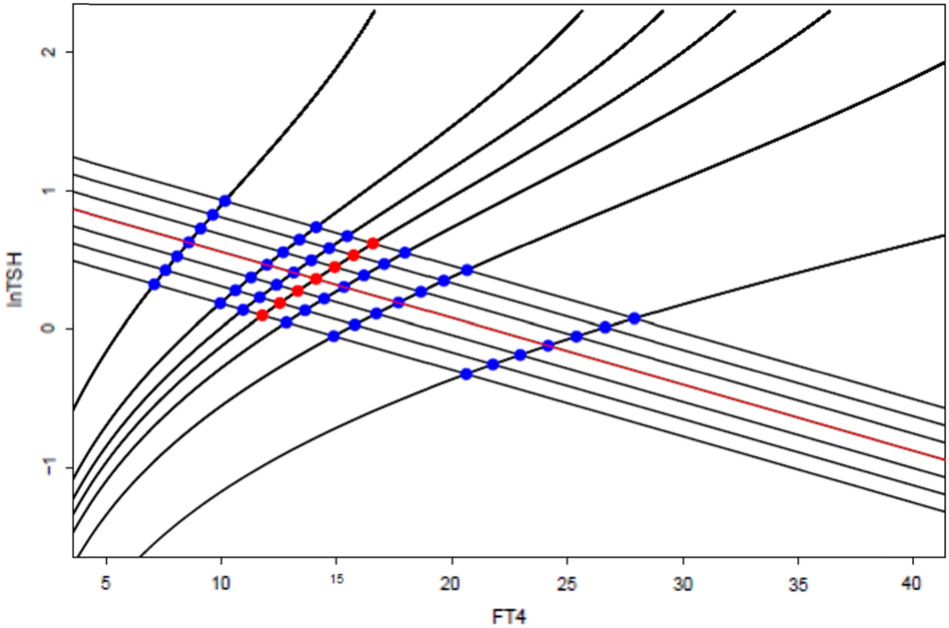
Diagrammatic representation of intersecting positively sloped FT4 and negatively sloped TSH curves generating a population distribution of FT4/TSH data points. The red line indicates the slope of the data corresponding to the slope of the TSH curve (Fitzgerald). The red data points indicate the 50th centile FT4 curve. It can be seen that each red data point is the 50th centile point on a TSH curve

We pursued the assumption that the population distributions of FT4 and TSH curves, as representing thyroid and pituitary sensitivities, were independent of each other and that therefore the slope of the data would approximate the slope of the TSH curve (Fig. 4).

Figure 4 demonstrates that the median FT4 curve is also theoretically plottable from the population FT4/TSH data by joining the median points on all of the TSH curves. Other percentile FT4 curves can be plotted analogously.

To analyse the data we used TSH zones rather than lines (TSH curves). TSH curves, oblique parallel lines, running on a negative slope that matched that of the data, starting from the middle line, were extended by a one-sided buffer zone each, using the functionality of the sf package [32], resulting in numbered parallel boxes or zones each 0.5 lnTSH units wide. This enabled us to calculate the median FT4 value in each zone. We put the zone numbers on the x- axis and the median FT4 values on the y-axis and performed a quantile regression over the zones to get the slope of median FT4 by increasing zone number.

As the zones are 0.5 lnTSH units each and equidistant the slope per zone equals the slope per 0.5 log units TSH. We multiplied this slope by 2 to derive the slope of FT4 over lnTSH. It should be noted that with the axes in their conventional configuration lnTSH is on the y-axis and FT4 is on the x-axis.

We performed analogous calculations to derive slopes for the 25th and 75th centile curves.

We performed various analyses of the population data with and without different methods of inclusion and exclusion of data points so as to exclude individuals with markedly abnormal thyroid physiology. We analysed the data restricted to the reference ranges of FT4 and TSH and also in terms of a range of normal FT4 and TSH curves as predicted theoretically. We used the ‘kite shaped’ distribution as described by Dietrich [1] and in addition plotted 2 other quadrilaterals (all plotted in log) defining other potentially normal distributions of FT4 and TSH curves. These various analyses served as sensitivity analyses as did analyses performed with variations in the slope of the zones.

On account our initial results we analysed the distribution of data within each of the TSH zones for any evidence of skew which might suggest the population data differed from theoretical predictions of independence predominantly by virtue of effects at the extremes of the data distribution. Such changes at the extremes might indicate the presence of physiological compensatory measures in individuals with FT4 levels at the upper and lower ends of the normal range. This analysis was performed by dividing each TSH zone into smaller sub-zones and examining the relative spread of data in these subzones. We analysed the distribution of data in the normal sample within a raster of 5 × 7 subzones, bounded by the minimum and maximum values of FT4 and lnTSH, respectively (contingency table of the raster, *p* = 0.87).

We then further analysed the data in terms of each individual’s calculated sensitivity to TSH and FT4. For TSH sensitivity, despite the previously mentioned limitations, we used the TSH Index (TSHI) and for FT4 sensitivity we used maximum thyroid output and maximum thyroid output/thyroid volume (GT and sGT). To calculate these parameters, we used previously published, validated formulae [27, 28].

## Results

All of our analyses indicated a physiology by which FT4 levels would only marginally change in response to a change in TSH levels. This translated into FT4 curve slopes (with FT4 on the y-axis) approximating 0 (see Table 1). By increasing the slope of our zones tenfold (and thereby introducing a discrepancy between the slope of the data and the slope of the zones) we still could only generate a slope of 1.46.

**Table 1:** Slopes of FT4 curves as calculated from 25th, 50th and 75th centile points

Data set	*n*	Restrictions	Data Slope	Slope of zones	Slope of 25th centiles	Slope of 50th centiles	Slope of 75th centiles
Normals	450	Nil	−0.05	−0.03	−0.10	−0.10	−0.13
Normals	448	Nil	−0.05	−0.05	0.10	0.33	0.20
Normals	448	Nil	−0.05	−0.25	1.0	1.2	1.3
Normals	446	Nil	−0.05	−0.50	1.44	1.42	1.46
Normals	439	Reference range	−0.05	−0.05	0.13	0.33	0.20
Normals	422	Reference range	−0.05	−0.12	0.4	0.70	0.6
EJE untreated	1424	Nil	−0.19	−0.20	0.12	0.17	0.15
EJE untreated	956	Reference range FT4/TSH	−0.06	−0.06	0.14	−0.06	0.04
EJE untreated	890	quadrilateral 1	−0.05	−0.06	−0.04	−0.18	−0.20
EJE untreated	1105	quadrilateral 2	−0.05	−0.09	−0.12	−0.25	−0.16
EJE untreated	784	quadrilateral 3 kite-shaped	−0.05	−0.05	−0.20	−0.04	−0.04
EJE untreated	799	quadrilateral 3 kite-shaped	−0.05	−0.25	−1.70	−1.72	1.97

We had considered a plausible slope would approximate the slope calculated by Dietrich et al. [1] and thereby approximate 10. With such a slope increasing the TSH from 0.3 to 3 mU/L would result in an increase in an increase in FT4 from 10 to 20 pmol/L. Therefore, in neither data set nor with any sensitivity analysis could we derive what we considered to be a plausible slope for the population FT4 curve.

**Fig. 5:**
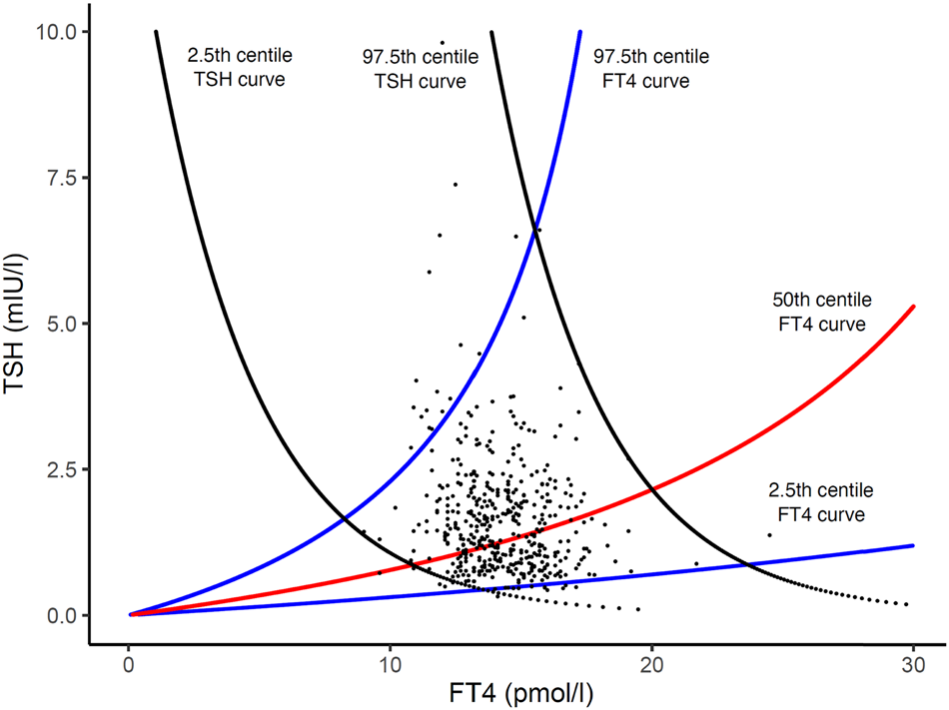
Data points superimposed on kite with highest and lowest TSH zones. It can be seen that adjacent the left negatively sloped TSH curve the data points are predominantly below the theoretical 50th centile FT4 curve i.e., a relative deficiency of data points at the most left part of this zone. The opposite pattern of data point distribution occurs at the other end of the kite, adjacent the right TSH curve. If the FT4 and TSH curves were independent the data points should be equally distributed above and below the 50th centile line at both ends of the kite as per Fig. 4

We concluded therefore that our premise of the random combination of FT4 and TSH curves was denied and that rather there was linkage between the curves in terms of their combination. We interpreted the finding of the slope of the calculated FT4 curve being too flat to indicate that this linkage resulted in a relative deficiency of data points at the low and high FT4 corners of the plot relative to the theoretical distribution of Dietrich et al. [1]. Indeed such deficiencies can be appreciated by simple inspection of a plot of the data overlying the theoretical distribution (Fig. 5).

The analysis of the distribution of data points within the different TSH zones, by examination of the subzones indicated no significant difference (chi-square test, *p* = 0.44). This suggested to us that the difference in the distribution of data between the normal data and the theoretical data could not be explained by changes at the extremes. The similar distribution of data in the subzones suggests that there has been a relative ‘shifting’ of data from a random pattern in the different TSH zones en bloc.

**Fig. 6:**
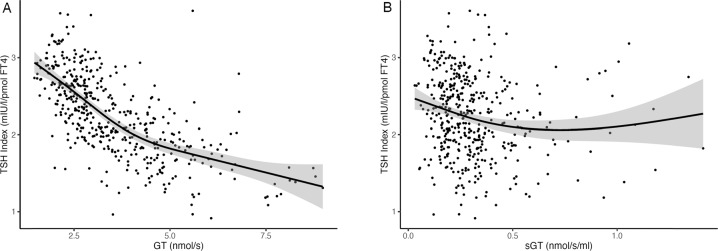
Plots of pituitary sensitivity to FT4 plotted against thyroid gland sensitivity as represented by TSH index plotted against GT (maximum thyroid output) (**A**), and SGT (GT/thyroid volume) (**B**). The shaded area surrounding the curve indicates the 95th confidence interval of the curve

This finding was confirmed in our analysis of GT versus TSHI which showed a strong negative relationship across the range (Fig. 6). We tested the negative slope of the relationship between GT and TSH index with a general additive model (*p* of the smooth term <0.001), with the data restricted to TSH values within the reference range. This negative range persisted but was attenuated when specific GT (sGT, GT/thyroid volume) was plotted against TSHI (p of the smooth term in the additive model <0.001) (Fig. 6). We interpreted this attenuation to confirm that the size of the thyroid gland contributes strongly to thyroid sensitivity to TSH [1].

## Discussion

Our inability to plot a plausible FT4 curve by reverse-engineering two large sets of population data of FT4/TSH pairs led to the finding of evidence of linkage in the combination of FT4 and TSH curves. We showed that thyroid glands with lesser responses to TSH in terms of FT4 output, i.e., less sensitive thyroids, tend to be paired with pituitaries with reduced response to FT4 inhibition of TSH output, i.e., less sensitive pituitaries. A reduced thyroid gland capacity to produce FT4 therefore not only results in an increased TSH level on account of a resulting low FT4 level; the TSH level is further increased on account of there also being an associated pituitary with lesser inhibition of TSH secretion for any level of FT4, than would be expected by chance alone.

**Fig. 7:**
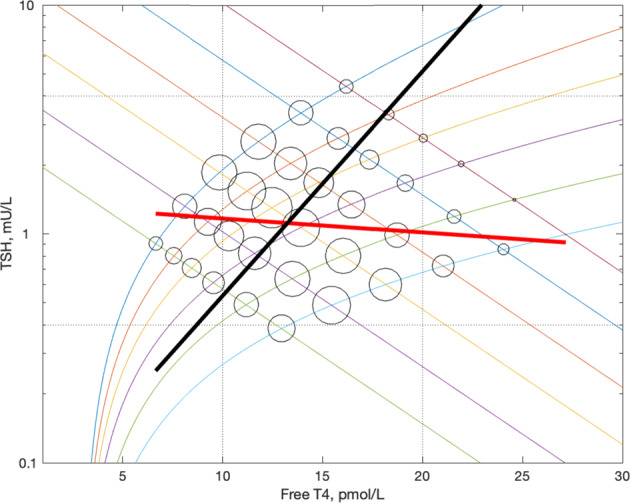
A theoretical distribution with dependence such that as we have demonstrated indicating the predicted discrepancies between the slope of the data and the slope of the TSH curves and between the actual slope of the FT4 curves and the slope of the FT4 curves as calculated by plotting the medians as we have done. Our results are consistent with the predictions

Figure 7 shows our previous theoretical generation of data with linkage affecting the combination of curves such that low FT4 output is associated with high TSH secretion. In agreement with our calculations it illustrates that like the TSH curve derived from population data [26], the FT4 curve as estimated from the line joining the median values in TSH zones, is rotated in an anti-clockwise direction as compared to the physiological curve.

Our results are consistent with those of Hoermann et al. [27] but by relying only on the assumption of a negative log-linear relationship between FT4 and TSH, by remaining consistent with multiple putative slopes of the TSH curve, and by being apparent with direct analysis of the data, are stronger. Our results do not depend on our confirmatory use of the TSHI which is again limited by its dependence on non-random combination of FT4 and TSH curves. The fact that the TSHI generates results that appear to be valid suggests any error in its use is relatively small.

Though a set point model of thyroid regulation would require linkage similar to what we have described [21], it would also imply, in contradiction of the empiric data [23, 24] a positive population FT4/TSH data slope [21].We propose three possible other mechanisms for the linkage affecting the combination of FT4 and TSH curves in individuals. These proposed mechanisms are not exclusive.

It may be that the normal population data results from the deletion of individuals with particular combinations of FT4 and TSH sensitivities bound to result in significantly abnormal thyroid function. This might occur through death in utero or exclusion from the data set by virtue of being on treatment. However, the fact that we found no difference between the data distribution in the different TSH zones, and that the negative relationship between GT and TSHI was present across the range suggests the TSH zones are shifted en bloc rather than there just being changes at the edges of the normal range.

Thus, it may be that pituitary and thyroid sensitivity are linked by common pathways. We cannot determine the mechanism of any such linkage between the thyroid and pituitary response curves from our data and this might be the topic of further research. We can hypothesize that there may be shared pathways determining the sensitivity of endocrine organs,i.e., there may be a shared mechanism resulting in both thyroid sensitivity to TSH stimulation and pituitary sensitivity to FT4 suppression. Another possibility might be restricted to just TSH sensitivity. At the thyroid level increased TSH sensitivity might increase thyroid output whereas at the pituitary level increased sensitivity to TSH might decrease TSH output secondary to increased autoregulation via short loop feedback [9, 10].

However, it is perhaps most likely that the linkage in the combination of FT4 and TSH curves reflects the response of thyroid and pituitary sensitivities to each other by virtue of trophic stimuli, e.g., in cases of low thyroid gland output higher TRH activity stimulates thyrotroph growth further increasing the TSH response. Such changes are well described in in pathological states [33]. Hoermann et al.’s data [25] also provided evidence for the activation of this physiological mechanism in the mildly abnormal state. We have previously suggested that such changes might be responsible for the shape of the population FT4/TSH correlation differing slightly from log-linear [26]. In these circumstances, thyroid and pituitary sensitivities, and thereby the FT4 and TSH curves, would be to some extent, directly inter-dependent.

Recent data on the HPA axis [34] has also shown that trophic effects and gland sizes are important factors in the regulation of hormones, generating feedback circuits with adjusted balance points to, e.g., coordinate seasonal patterns. In these circumstances however additional factors beyond the feedback loops may contribute to any trophic changes.

Our findings extend our previous conclusion [26] that the population curves for TSH and FT4 need not resemble the physiological curves, to the conclusion that they cannot be similar. The similarity between our previously chosen examples of experimentally derived and population data derived TSH curves was interpreted as an indication of independence in curve combination [26], but in retrospect we note that the authors of the paper reporting the relevant experimentally derived estimate of the TSH curve did in fact indicate that they suspected their estimated slope was too flat [16].

Our finding of linkage weakens our previous conclusion regarding the greater inter-individual variation of FT4 curves as compared with TSH curves. Though in the presence of random combination of FT4 and TSH curves a negative population slope implies greater variation in FT4 curves, the presence of linkage may lead to a negative population slope in the presence of equal or greater inter-individual variation in TSH curves, providing any excess is not too great.

Our work indicates that on account of the demonstrated linkage in curve combination, the proposed methods of examination of population data to date result in underestimation of the slopes of the FT4 and TSH curves. It follows that further physiological studies are required to better understand this physiology. The physiological studies thus far concern the TSH curve only and the results are difficult to interpret on account of the use of different methodologies and different assays and parameters of thyroid function [16–19].

Further physiological studies however will need careful design. Given that there may be continuous interaction between thyroid and pituitary sensitivities the corresponding curves may not be fixed, and results may therefore vary according to experimental design. Thus, precise plotting of the FT4 and TSH curves of individuals in a population would be a formidable undertaking and may not even be possible now. Such studies may however generate data that is sufficiently accurate to allow direct testing of our conclusions which remain based on indirect analysis.

The linkage in the combination of FT4 and TSH sensitivities may also indicate evolutionary pressures. The FT4/TSH feedback loop remains the dominant regulator of thyroid hormone levels. This feedback loop responds to acute perturbations in thyroid physiology. Any subsequent adjustments of the feedback loops can result in a slower augmentation of this homeostatic response if the stimulus for any response persists. The linkage we demonstrated acts in response to primary variations in thyroid function thus to homeostatically stabilise FT4 values in the individual but also to minimise the spread of FT4 values in the population, maximising the chances of each individual having mid-range levels. As there is evidence that pathology increases with deviation of thyroid hormone levels in either direction [11] from the midzone, minimising such intra- and inter-individual spread would be evolutionarily advantageous.

This work shows that the normal regulatory feed-back physiology of FT4 and TSH is more complex than previously proposed [1] in that normal physiology cannot be inferred solely by reference to FT4 and TSH curves which individually are normal. Rather, there are particular combinations of FT4 and TSH curves which reflect normal physiology. These normal combinations of curves result in FT4 levels reasonably close to the middle of the population range, levels suitable for euthyroid peripheral organ function. This physiology cannot be represented by a simple rectangle or kite.

In summary, the pattern of the population FT4/TSH relationship not only provides evidence that there is a balance point rather than a set point for thyroid function [21], but also provides evidence of linkage of the sensitivities of the thyroid and the pituitary glands in terms of their combination in individuals. This linkage is a barrier to deriving the curves describing these sensitivities from this same data or from physiological experimentation, but it may be evolutionarily advantageous and contribute to homeostasis.

This paper provides a further advance in the understanding of the TSH-FT4 relationship, which is central to thyroid physiology and pathophysiology, and laboratory diagnosis of thyroid disease [24]. Further studies might indicate whether or not the described principles apply in other systems such that they have fundamental importance generally in endocrinology and homeostasis. The simplicity of our method, not requiring complex formulae and definitions regarding the sensitivity of the relevant feedback loops, may be a particular advantage in the study of other systems in which such formulae and definitions have not been so developed.
